# Differing requirements for Augmin in male meiotic and mitotic spindle formation in *Drosophila*

**DOI:** 10.1098/rsob.140047

**Published:** 2014-05-14

**Authors:** Matthew S. Savoian, David M. Glover

**Affiliations:** Department of Genetics, University of Cambridge, Downing Site, Cambridge CB2 3EH, UK

**Keywords:** division, γ-tubulin, microtubule, spermatocyte, kinetochore, nucleation

## Abstract

Animal cells divide using a microtubule-based, bipolar spindle. Both somatic, mitotic cells and sperm-producing male meiotic spermatocytes use centrosome-dependent and acentrosomal spindle-forming mechanisms. Here, we characterize the largely undefined, centrosome-independent spindle formation pathway used during male meiosis. Our live and fixed cell analyses of *Drosophila* spermatocytes reveal that acentrosomal microtubules are nucleated at kinetochores and in the vicinity of chromatin and that together these assemble into functional spindles. Mutational studies indicate that γ-tubulin and its extra-centrosomal targeting complex, Augmin, are vital for this process. In addition, Augmin facilitates efficient spindle assembly in the presence of centrosomes. In contrast to the pronounced recruitment of Augmin on spindles in other cell types, the complex is absent from those of spermatocytes but does accumulate on kinetochores. Polo kinase facilitates this kinetochore recruitment while inhibiting Augmin's spindle association, and this in turn dictates γ-tubulin distribution and spindle density. Polo's negative regulation of Augmin in male meiosis contrasts with its requirement in loading Augmin along mitotic spindles in somatic *Drosophila* cells. Together our data identify a novel mechanism of acentrosomal spindle formation in spermatocytes and reveal its divergence from that used in mitotic cells.

## 2. Introduction

Chromosome segregation and genome partitioning in animal cells require the assembly of a microtubule (MT)-based bipolar spindle. In centrosome-containing mitotic cells, spindle formation is driven by two complementary processes. In the first, astral MTs nucleated at each of the two centrosomes probe the former nuclear volume until their dynamic plus ends contact and are bound by centromere-associated kinetochores through a process of ‘search and capture’. The second, acentrosomal means appears to act through multiple pathways and employs the nucleation of MTs beyond the centrosomes often near the chromosomes [1–5]. Although centrosomal and acentrosomal mechanisms are contemporaneous, they can be experimentally uncoupled, e.g. by deactivating the centrosomes through laser microsurgery [6] or genetic manipulations [7–9], without precluding bipolar spindle formation.

Augmin is a recently identified contributor to centrosome-independent mitotic spindle assembly. This complex serves a conserved role in fly and vertebrate cells where it targets γ-tubulin and its MT nucleating γ-tubulin Ring Complex exclusively along the spindle. Augmin assumes a similar distribution and likewise also collects at the centrosomes [10–21]. Depletion of any of the Augmin complex's eight subunits results in a destabilization of the others and dramatically reduces spindle MT density. As a result, chromosome alignment and segregation become perturbed [11,13,14,16–22]. These defects are compounded when the centrosomes are removed or pre-existing MTs are depolymerized and allowed to regrow [11,14,21]. In such instances, few MTs appear around the chromosomes and robust spindles fail to form. These phenotypes, in combination with recent direct observations of Augmin-dependent MT branching [12], identify the complex as a crucial governor of mitotic spindle integrity by mediating acentrosomal MT nucleation.

Comparatively little is known about the mechanisms responsible for spindle assembly during male meiosis. Consistent with the presence of centrosomes, studies in insect spermatocytes indicate that these cells also use a centrosomal MT ‘capture’ pathway [23–26]. Yet in *Drosophila*, anchoring of the centrosomes to the plasma membrane [26] or incapacitating the centrosome through mutation [27] does not preclude spindle MT generation. Some of the resultant MTs can be traced back to the persisting nuclear membranes that surround the spindle and that are characteristic of *Drosophila* spermatocytes, while others appear to be ‘nucleoplasmic’ [26–28]. The ability of male meiotic cells to establish MT structures independently of centrosomes is further demonstrated by the generation of MTs in association with chromosomes that have been mechanically placed within the cytoplasm [29]. Irrespective of the experimental means used to produce these acentrosomal spindles, they are at least partially functional and support the separation and segregation of chromosomes [26,29].

The molecular understanding of this acentrosomal pathway is rudimentary. Augmin has only been reported to localize to spermatocyte centrosomes [21]. Inactivation of the complex by mutation of its Wac subunit does not alter spindle morphology in fixed preparations and aneuploidy only slightly increases [14]. The relative roles of the γ-tubulin Ring Complex in the two spindle formation pathways is also not clear and understanding is further obscured by the differences between cell types. In mitosis, downregulation of either γ-tubulin or the dd4 subunit precludes aster formation, suggesting loss of centrosome function, and yet does not prevent bipolar spindle assembly. Conversely, mutant spermatocytes assemble astral arrays but their spindles collapse over time [30–33]. These observations suggest that despite their outwardly similar appearance, male meiotic and mitotic spindles form through divergent mechanisms leading us to characterize acentrosomal spindle assembly in spermatocytes. We find that, like mitotic cells, spermatocytes form functional acentrosomal spindles in an Augmin and γ-tubulin-dependent manner. However, the distribution of these complexes and their regulation in male meiosis differ from mitosis, pointing towards an alternative mechanism for acentrosomal MT nucleation and spindle formation.

## 3. Results and discussion

### 3.1. Spermatocytes employ centrosomal, acentrosomal and Augmin-mediated spindle formation pathways

**Figure 1.**
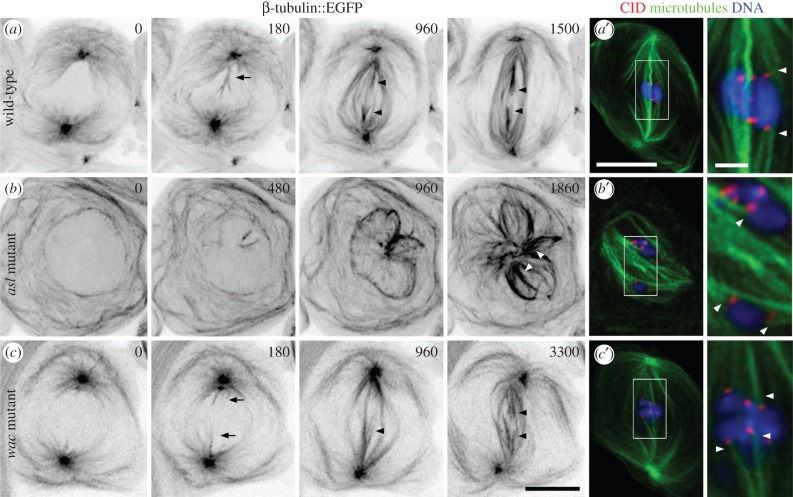
Meiotic spindles form through centrosomal, acentrosomal and Wac-mediated pathways. (*a–c*) Time-lapse sequences of spindle formation in the indicated genetic backgrounds visualized using β-tubulin::EGFP expression. (*a*) Wild-type spindle formation initiates as centrosome-nucleated astral MTs penetrate into the nucleus (180; arrow). These increase in number and organize into bundles (180–960), some of which are k-fibres (arrowheads) as revealed by their contact with chromosome-based fluorescence ‘ghosts’. The chromosomes align at the spindle equator where they remain at metaphase (1500), shortly before anaphase entry. (*a′*) Fixed wild-type metaphase cell showing the distribution of the centromeric protein CID/CENP-A as a marker for kinetochore position, MTs and DNA. Arrowheads denote k-fibres. (*b*) A centrosome inactivated, *asl^2^/asl^3^* mutant. Neither centrosomes nor asters are detected. Spindle assembly is first observed with the nucleation of a few MTs within the nucleus. Progressively more MTs appear that interact (480–960) to establish larger structures. These are poorly organized, but exhibit k-fibre-like MT bundles (1860; arrowheads). (*b′*) Fixation and staining of *asl^2^/asl^3^* cells confirms that k-fibres (arrowheads) form in the absence of centrosomes. (*c*) Spindle assembly in a *wac Δ12* hemizygous mutant. Like the wild-type, centrosomal MTs invade the nucleus (0–180; arrows). However, these require protracted periods to organize into a spindle with recognizable albeit non-robust k-fibres (960; arrowhead). As the k-fibres mature (arrowheads) the chromosomes assume an equatorial position where they remain throughout metaphase (3300). (*c′*) Fixation and staining of *wac* mutant cells confirms that this protein and by extension the Augmin complex are not needed for k-fibre formation (arrowheads) or normal spindle morphology. Time is in seconds relative to the onset of spindle formation. All images are z-projections. Bars are 10 μm except in zoomed fixed images where they are 2 μm.

We began our examination of spermatocyte spindle formation by characterizing the contributions of the centrosome. For this, we performed time-lapse imaging of spermatocytes expressing β-tubulin56D::EGFP (enhanced green fluorescent protein) to label MTs [34]. In wild-type cells, spindle formation initiates at prometaphase as centrosome-derived astral MTs penetrate into the former nuclear volume—a compartment that remains partially delineated throughout division by the persisting layers of membrane associated with the nuclear envelope (figure 1*a*; electronic supplementary material, video S1) [23,24,28]. At this time, the chromosomes, often detectable as ‘ghosts’ against the background fluorescence, begin to move. Progressively more MTs extend from the polar regions and organize into kinetochore fibres (k-fibres; arrowheads), bundles that link each kinetochore to the spindle. This process repeats until all of the chromosomes align at metaphase (figure 1*a*′). We next examined MT generation in the acentrosomal pathway by studying mutants trans-heterozygous for *asterless* (*asl^2^/asl^3^*), which encodes a centriolar protein. Fixed preparations of these cells previously revealed that they lack centrosomes but retain the ability to build spindle-like structures [27]. When we examined living cells, we found disorganized MTs throughout the cytoplasm and, following prometaphase onset, also in the nucleus where they surrounded the chromosomes. The latter MTs increased in density and became loosely organized over time to form multi-polar spindle-like structures (*n* = 10 cells; figure 1*b*; electronic supplementary material, video S2). Although we were unable to assess the functionality of these formations, at least some of their MTs assembled into k-fibres (figure 1*b,b′*; arrowheads). Together our data confirm that spermatocytes engage centrosome and centrosome-independent MT nucleation and spindle assembly mechanisms.

We next investigated the effects of Augmin inactivation using the same Wac subunit mutant previously examined in fixed preparations [14]. The *wacΔ 12* lesion excises most of the gene and when placed over a deficiency virtually no protein is detected [14]. Our time-lapse analyses revealed that *wac* hemizygotes took on average 55% longer to enter anaphase than controls (56 ± 1 min, *n* = 13, versus 36 ± 2 min, *n* = 11, respectively). As shown in figure 1*c* (electronic supplementary material, video S3), this lag was due to a delay in spindle formation. Although astral MTs penetrated into the former nucleus as in the wild-type, more time was required before substantive k-fibres could be detected. Once established, spindles in the mutants appeared largely normal (figure 1*c′*; arrowheads). Thus, Wac and by extension the Augmin complex are needed for efficient meiotic spindle formation and a timely anaphase onset. This role does not appear to be performed at the centrosomes (see below).

### 3.2. Acentrosomal spindle microtubules are nucleated at multiple nuclear sites including kinetochores

Having shown that male meiosis can use centrosome-independent spindle assembly mechanisms, we defined the sites of acentrosomal MT nucleation. MT re-growth was studied in spermatocytes treated with the photo-labile MT depolymerizing agent colcemid. MT-free pre-anaphase cells were identified and followed by time-lapse imaging prior to being pulsed with UV light to inactivate the drug and initiate MT polymerization. By using cells expressing EGFP-tagged tubulin to label MTs and Aurora B::mCherry to visualize the centromeres and demarcate the kinetochores, we were able to exclude those cells with incompletely depolymerized spindles and unambiguously track the origins of newly formed MTs.

**Figure 2.**
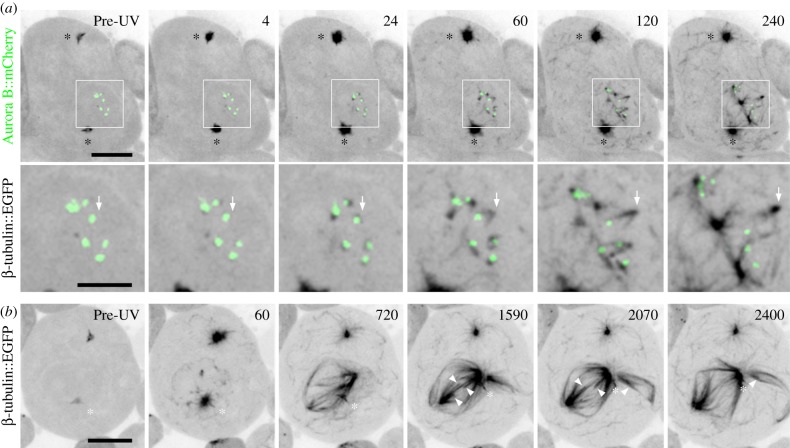
Functional acentrosomal spindles form from MTs nucleated within the nucleus and at kinetochores. (*a*) Sequence showing MT regrowth in a wild-type spermatocyte expressing both β-tubulin56D::EGFP and Aurora B::mCherry to label MTs and kinetochore position, respectively. Prior to the UV pulse, no MTs are detected in the low magnification or boxed and zoomed panels. After the pulse, MTs emanate from the centrosomes (*) and form throughout the cytoplasm. Within the nucleus MTs appear at the persisting membranes, in the nucleoplasm and directly adjacent to the kinetochores. These latter MTs first appear as foci but rapidly assume a ‘v’ shape as they extend away from the kinetochore led by their vertices (arrows follow formation and extension of a single example). Non-kinetochore nucleated MTs undergo similar movements. Both populations can fuse into larger more intricate structures. (*b*) Sequence from an increased duration recording of an EGFP-tagged tubulin expressing cell following MT regrowth. In this cell, a functional, multi-polar spindle forms. Although one centrosome is proximal to the nucleus (*), it does not appear to contribute substantial numbers of MTs and instead travels along the acentrosomal half-spindle towards its pole. As the spindle matures (1590), k-fibres are observed (arrowheads) which eventually shorten (2070–2400) similar to those in anaphase controls. Time is in seconds relative to the UV pulse. All images are z-projections. Bars are 10 and 5 μm in low magnification and zoomed panels, respectively.

Following the UV pulse, MTs appeared in multiple cellular regions: at the centrosomes, within the cytoplasm, and around and within the former nucleus (*n* = 12 cells; figure 2*a*). As noted previously [26], some of these nuclear MTs re-grew off of the membranes surrounding the nuclear compartment. We further observed prominent nucleation in this nucleoplasm and directly at the kinetochores. The maturation of one kinetochore-derived MT structure is detailed in figure 2*a* (arrows; electronic supplementary material, video S4). We found that nucleation initiated rapidly, and within 4 s of the UV pulse tubulin foci appeared directly adjacent to 80% of the Aurora B signals (*n* = 58 kinetochores). Kinetochore-nucleated MTs assumed a short half-spindle-like ‘v’ shape that elongated and moved away at 1.1 ± 0.2 μm min^−1^ (*n* = 16), often coalescing into more elaborate structures. Imaging of EB1::EGFP and Aurora B::mCherry indicated that, as with control centrosome-derived spindles, kinetochore-nucleated half-spindles were polarized with their minus ends focused into poles and their dynamic plus ends nearest to the chromosome (electronic supplementary material, figure S1). It is noteworthy that a similar population of short kinetochore-bound MTs has also been detected by electron microscopy in wild-type prometaphase [23,24]. Our live cell work now accounts for the origin these naturally occurring MTs and further reveals that they can promote spindle assembly. The v-shaped morphology of meiotic kinetochore-derived MT structures varies from those reported for mitosis, which appear as a single unified bundle (e.g. [21,35,36]). It is possible that this difference reflects the geometry of the source kinetochores. Unlike mitotic cells, each primary spermatocyte kinetochore is a compound structure composed of two partially resolved and closely positioned sister chromatid kinetochores [37]. We propose that MTs are independently nucleated at each of these adjacent sites. These elongate and rapidly organize and fuse together in a manner analogous to that of the nucleoplasmic MTs described earlier and similar to the motor protein-mediated mechanisms reported for mitosis [35,38,39].

To assess the functionality of regrown spindles, 16 cells were followed for a 40 min interval, in which time control centrosomal spindles always entered anaphase. During this regrowth period, bi- or multi-polar spindles repeatedly assembled as a result of the interactions of nucleoplasmic, kinetochore and in some instances centrosomal MTs (figure 2*b*; electronic supplementary material, video S5). Approximately 50% of these cells proceeded to enter anaphase and display shortening k-fibres and elongating spindles similar to MT behaviour in control cells (figure 2*b*; arrowheads). In conclusion, we find that functional acentrosomal spindles are formed in spermatocytes through the coalescence of MTs nucleated at kinetochores and other nuclear sites. These structures do not appear to be inhibited or stimulated in the presence of the centrosomes and promote timely anaphase onset and chromosome segregation.

### 3.3. Augmin collects at meiotic kinetochores but only weakly decorates the spindle

**Figure 3.**
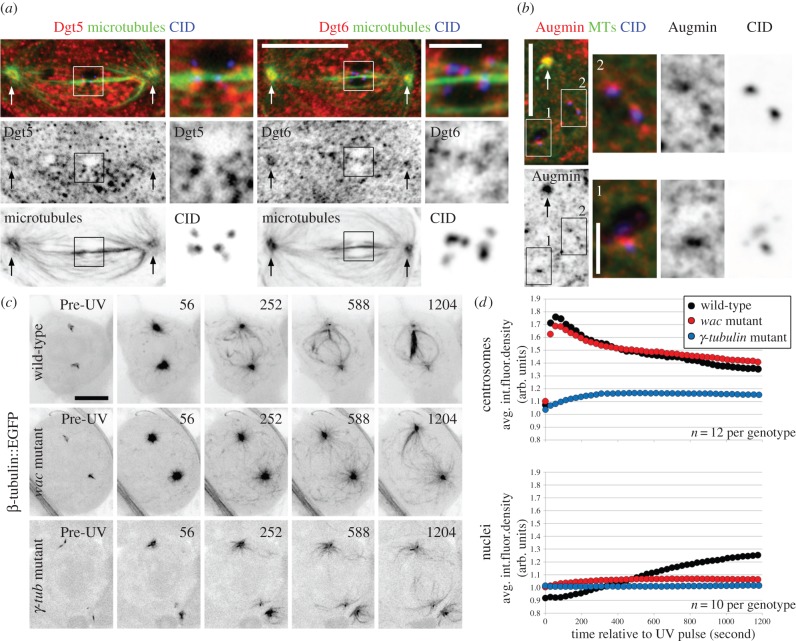
Augmin decorates meiotic kinetochores but not spindles and promotes nuclear MT formation. (*a*) Augmin distribution in metaphase spermatocytes as shown by Dgt5 and Dgt6 staining. Augmin localizes to the centrosomes (arrows) but does not concentrate on the spindle's MTs. Zoomed panels reveal that Augmin forms foci at the centromeres (CID). (*b*) MTs depolymerization does not prevent recruitment of Augmin to the centrosomes (arrows) or at centromeres (corresponding zoomed panels) indicating that it is a kinetochore component. (*c*) Sequences showing MT regrowth in wild-type, *wacΔ 12* hemizygous or *γ-tubulin23C^pi^* homozygous mutants, each expressing β-tubulin::EGFP. The *wac* mutation does not affect nucleation in the cytoplasm or the activity of the centrosomes while severely hindering nuclear MT formation. Downregulation of γ-tubulin compromises all MT nucleation events. After a delay, only a few MTs appear at the centrosomes or in the nuclear region. Time is in seconds from the UV pulse. (*d*) Kinetic profiles of average tubulin fluorescence density at the centrosomes and within the nuclei of wild-type, *wac* and *γ-tubulin* mutant cells during MT regrowth. Fluorescence quantification is in arbitrary units (arb. units). The plots confirm Wac's nucleus-confined functions and the necessity of γ-tubulin in MT formation throughout the cell. See text for details. All images except (*a*) are z-projections. Bars are 10 μm except in zoomed panels where they are 2 μm.

Our live cell observations of *wac* mutants indicated that Augmin is important for meiotic spindle formation. Because no detailed study of Augmin in male meiosis has been reported, we began characterizing the contributions of the complex by defining its localization using antibodies against two of its subunits, Dgt5 [11] and Dgt6 [21] (figure 3*a*). As expected from their interaction as part of the hetero-octamer, the two proteins show indistinguishable immuno-localizations, and we subsequently refer to them interchangeably as Augmin. Staining of metaphase cells was marked by a high background with punctae throughout the cell. Despite this, the signal sometimes appeared elevated in the region of the spindle. Although we noted some recruitment to the centrosomes as previously reported [21] (figure 3*a*; arrows), we were unable to detect any distinct co-localization with the k-fibres or other MTs. This staining pattern differs from the pronounced and homogeneous decoration of the spindle by Augmin that is characteristic of mitotic metaphase in vertebrate [13,18,20,40,41] and *Drosophila* tissue culture cells ([10,11,14,17,21]; see below). It also differs from the polar association found in *Drosophila* meiotic oocytes [14,42]. We consistently observed Augmin foci proximal to all of the centromeres we examined (*n* = 80). However, the signal could be difficult to discern from the background of nearby nucleoplasmic granules. We therefore treated cells with colcemid to depolymerize MTs and determine if the Augmin accumulations were at kinetochores, on MT ends or adjacent aggregates. We found that Augmin was present at centrosomes and centromeres in all cases (*n* = 80; figure 3*b*). Augmin has also been previously reported to bind to mitotic kinetochores in *Drosophila* tissue culture cells. In contrast to our findings, those studies indicated that kinetochore association is MT-dependent [21]. We do not think that our kinetochore localization following colcemid treatment is due to the retention of depolymerization resistant MTs, as Augmin is also detected in early prometaphase cells prior to the onset of spindle assembly (electronic supplementary material, figure S2). From these observations, we conclude that Augmin exhibits a male meiosis-specific distribution. Unlike the other systems examined to date, it is not enriched on any region of the spindle. Furthermore, it binds kinetochores irrespective of MT attachment status. These data suggest specialized functions and regulatory mechanisms in this cell type.

### 3.4. Augmin and γ-tubulin are required for microtubule nucleation and acentrosomal spindle formation

To directly assay whether Augmin contributes to acentrosomal spindle formation, we carried out MT regrowth assays in *wac* Δ12 hemizygotes expressing β-tubulin56D::EGFP. While MT nucleation appeared unaffected at the centrosomes and in the cytoplasm, it was severely compromised in nuclei. MTs were largely absent from this region and most of the nuclear MTs appeared to emanate from the centrosomes (*n* = 14 cells; figure 3*c*; electronic supplementary material, video S6).

**Figure 4.**
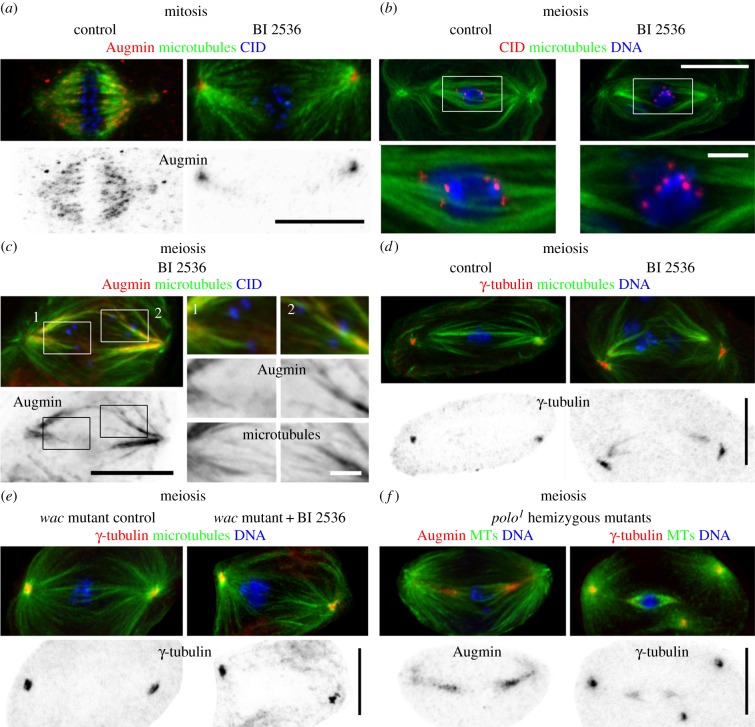
Polo kinase differentially regulates Augmin distribution and spindle morphology in mitosis and male meiosis. (*a*) Comparison of spindle morphology in control and BI 2536 Plk1 family-specific inhibitor treated mitotic tissue culture cells. In control cells, Augmin homogeneously coats the spindle and localizes to the centrosomes. BI 2536 treatment results in a characteristic loss of k-fibres and spindle attachment as revealed by MT and CID distribution. Augmin is almost entirely excluded from the spindle but remains at the centrosomes. (*b*) Identical BI 2536 treatment of spermatocytes leads to increased MT density near the spindle poles with a concomitant loss of k-fibres causing the chromosomes to lay loose on the spindle (zoomed panels). (*c*) BI 2536 treatment mis-localizes Augmin in spermatocytes. The complex becomes aberrantly recruited to the MT-rich portions of the spindle. The signal is greatest near the poles and extends towards the equator. It is no longer observed at the centrosomes or kinetochores (CID; zoomed panels). (*d*) Polo inhibition alters γ-tubulin distribution. BI 2536 treatment redirects the protein from the centrosomes to the polar regions corresponding to the enhanced MT density. (*e*) Wac activity is required for BI 2536-mediated spindle and localization phenotypes. Treatment of *wac* mutants with BI 2536 does not dramatically increase spindle MT numbers or affect γ-tubulin's centrosome exclusive placement. (*f*) Augmin and γ-tubulin distribution following Polo downregulation in *polo^1^* hemizygous mutants. As with BI 2536-treated spermatocytes, Polo inactivation through mutation results in Augmin and γ-tubulin assuming pronounced spindle distributions that extend from the poles towards the equator. See text for details. All images are z-projections. Bars are 10 μm except in zoomed panels where they are 2 μm.

If as in mitosis [10–12,14,16–19,21] Augmin functions in spermatocytes as an extra-centrosomal γ-tubulin targeting factor, then reduced Wac or γ-tubulin should lead to overlapping phenotypes. We found that loss of Wac did not prevent γ-tubulin23C, the sole γ-tubulin isoform expressed in testes [43], from accumulating at the centrosomes (electronic supplementary material, figure S3*a*; figure 4*e*); nor did the depletion of γ-tubulin preclude Augmin from loading at this now diminished structure (electronic supplementary material, figure S3b). When challenged in the MT re-growth assay the *γ-tubulin* mutants behaved like those downregulated for Wac with only feeble numbers of MTs appearing within the nuclear compartment. In addition, few MTs appeared at any cellular location including the centrosomes (*n* = 12 cells; figure 3*c*; electronic supplementary material, figure S6). The extent of this reduction in MT generation was surprising given that mutants of *γ-tubulin* and other core components of the MT nucleating γ-tubulin Ring Complex routinely form astral MT arrays of normal appearance that cap the ends of unstable but bipolar spindles [30,33] (electronic supplementary material, figure S4a). We find that spindle and kinetochore interactions are regularly obscured in these mutants. K-fibres, which in wild-type cells appear as MT bundles terminating at the kinetochore, are often difficult to detect, while kinetochores commonly contact an MT's lateral surface (electronic supplementary material, figure S4b). Thus, although untreated *γ-tubulin* mutants display large numbers of MTs, their nucleation potential and ability to form functional spindles is severely compromised.

To quantitatively define Wac's contribution to spermatocyte spindle formation, we measured the changes in tubulin fluorescence that occur during MT regrowth (figure 3*d*). In the wild-type, centrosome fluorescence rapidly peaked to a maximum intensity within approximately 60 s of the UV pulse. This value then declined in a biphasic manner possibly due to the shedding of nucleating components. Nuclear MT formation was kinetically distinct as shown by the slow and predominantly linear increase in fluorescence following an approximately 100 s initial lag. Although nuclear fluorescence intensity increased throughout the course of filming, it never exceeded that of the centrosomes. Thus, in spermatocytes the centrosome is the kinetically favoured site of MT nucleation. Recapitulating our qualitative assessments, loss of Wac function did not diminish the rate or extent of centrosome nucleation; the mutants' fluorescence profile was virtually indistinguishable from that of the wild-type. Conversely, nuclear fluorescence became only slightly elevated, in accord with a severely dampened ability to generate spindle MTs acentrosomally. The greatest decrease in MT nucleating potential followed γ-tubulin depletion. The centrosomes in *γ-tubulin* mutants did not exhibit the burst of nucleation seen in either of the other lines. Instead, the fluorescence intensity reached its maxima over approximately 300 s, a period in which wild-type and *wac* mutant centrosomes' fluorescence had already begun to wane. Acentrosomal nucleation was almost entirely abolished in both *wac* and *γ-tubulin* mutants and nuclear fluorescence showed almost no change over time (figure 3*d*). Together our data indicate that although Augmin is found at centrosomes, it does not govern astral MT nucleation. It is however a vital determinant of acentrosomal MT nucleation specifically within the nuclear compartment. In addition and despite earlier reports, γ-tubulin is required for MT nucleation in all cellular compartments. This findings are consistent with Augmin performing a γ-tubulin targeting function outside of the centrosome.

Our collective observations strongly implicate Augmin as a critical component of the male meiotic spindle assembly machinery. While we cannot rule out that a small pool of the complex acts in the surrounding nucleoplasm or along the length of pre-existing MTs as occurs in mitosis, our localization and regrowth data are most consistent with Augmin driving spindle formation largely at the kinetochore. We envisage this occurring, first, through the nucleation of MTs which, as they extend outwards from this structure, increase the area available to contact and capture invading astral MTs in a process similar to that of mitosis [35]. Second, by associating with a complex of proteins including factors belonging to the XMAP215/Msps family [21], Augmin complexes promote the stabilization and subsequent integration of these newly captured MTs into k-fibres for faithful spindle attachment.

### 3.5. Polo kinase positively and negatively regulates Augmin distribution for microtubule nucleation and acentrosomal spindle formation

As Augmin has a unique distribution in spermatocytes, this raised the question of whether or not its localization was governed by similar mechanisms to those regulating its localization in mitosis. Recent work in vertebrate mitotic cells has identified Polo-like kinase 1 (Plk1) as a positive regulator of Augmin's spindle recruitment [19,40]. To determine whether *Drosophila* uses a similar regulatory pathway, we examined Augmin distribution in mitotic tissue culture cells following Polo inactivation using BI 2536, a small molecule inhibitor specific for the Plk1 family [44,45]. As with earlier reports [11,12,14,17,21], control mitoses were always characterized by an Augmin signal that collected at the centrosomes and intensely labelled the full length of the spindle, accentuating the k-fibres (*n* = 40 cells). When cells were briefly incubated with BI 2536, both spindle morphology and Augmin localization became aberrant. Bipolar spindles became elongated and the asters now extended towards the equator. K-fibres were absent although lateral contacts between MTs and kinetochores were sometimes visible [44]. Invariably, Augmin still formed foci at the centrosomes but now only weakly associated with the polar-most parts of the spindle (figure 4*a*; 40/40 cells). Thus, as with vertebrate cells, Polo kinase positively governs Augmin's extensive deposition on *Drosophila* mitotic spindles.

As the first step in defining Polo's Augmin-regulating role in male meiosis, we determined how BI 2536 treatment might affect meiosis in spermatocytes. We found that, similar to mitotic cells, BI 2536 treatment of spermatocytes caused a loss of k-fibres with the chromosomes assuming a centralized but poorly ordered position (figure 4*b*). These abnormal spindles were further marked by increased MT numbers near the poles but few at the equator. We then studied the effect of Polo inactivation on Augmin by studying the distribution of Dgt5 and Dgt6. Drug treatment led to the identical mis-localization of both of these subunits. Unlike controls where Augmin resides at centrosomes and kinetochores but only feebly localizes to the spindle, Polo inhibition resulted in a decreased signal at centrosomes, a loss of the complex from kinetochores (*n* = 50) (cf. figures 3*a* and 4*c*) and, strikingly, extensive accumulation on the spindle. The signal was maximal near the poles and extended by variable amounts towards the equator (figure 4*c*). This ectopic placement was associated with the increased numbers of polar MTs and suggested enhanced acentrosomal MT nucleation. Indeed, whereas γ-tubulin is normally detected only at meiotic centrosomes it now also labelled the spindle's polar regions (figure 4*d*). Both changes were Augmin-mediated as BI 2536 treatment failed to alter γ-tubulin distribution or increase MT density in Wac-depleted cells (figure 4*e*). These data indicated that Polo both positively and negatively regulates different aspects of Augmin distribution and spindle morphology. To corroborate such unexpected findings, we examined Augmin and γ-tubulin distribution following Polo downregulation in *polo^1^* hemizygous mutants [46]. Despite the weak penetrance of this mutation, both proteins still became abnormally positioned along the spindle (figure 4*f*). Therefore, Polo promotes Augmin's placement at kinetochores while preventing its association with spindle MTs. This inhibitory function limits γ-tubulin distribution with a corresponding regulation of spindle MT numbers.

It is unclear whether Polo acts directly on male meiotic Augmin complex members or whether it controls localization through additional factors. In *Drosophila* mitosis, Augmin's Dgt6 subunit directly binds to Ndc80 [21]. Studies on both mitotic fly [47] and vertebrate [48] tissue culture cells reveal that Polo/Plk1 inhibition or downregulation reduces Ndc80 levels at the kinetochore. We also observe a reduction of Ndc80 at spermatocyte kinetochores after BI 2536 treatment (M.S.S. & D.M.G. 2014, unpublished data). Thus, the loss of Augmin that we report can be explained, at least in part, by a requirement for Polo to regulate kinetochore composition through other substrates.

We are unaware of similar precedents to account for our finding that Polo inhibits Augmin's spindle association in spermatocytes. Our experiments in mitotic fly cells recapitulate those from vertebrates where, in both systems, Polo/Plk1 positively regulates the homogeneous distribution of the Augmin complex along the spindle's length. In mitotic vertebrate cells, spindle loading requires the phosphorylation of Augmin's MT binding subunit, Hice1. Disruption of this modification prevents Augmin recruitment, and MT nucleation is diminished [20,40]. To the best of our knowledge, no data have been presented on the phosphorylation state of any of Augmin's subunits in *Drosophila*. However, an alternative regulatory scheme must operate, because *Drosophila* lacks a Hice1 homologue [20]. Some insights may be gained from *Drosophila* meiotic oocytes, where Augmin neither homogeneously coats the spindle nor is entirely excluded from it. Rather, the complex is spatially confined to the spindle poles, where it controls local MT density for chromosome alignment [14,42]. While no mechanism has been described to account for this distribution, studies of fluorescence recovery after photobleaching reveal there are two pools of Augmin in the oocyte, both of which are more dynamic than reported in mitotic cells [11,42]. This raises the possibility that male meiotic cells might also have a small, unstable Augmin population that transiently associates with the spindle. Polo inhibition may alter this dynamism either directly by acting on complex members or indirectly through unidentified accessory factors, thereby arresting the pool in a spindle-bound state and resulting in the mis-localization we report. It will therefore be of great interest to determine Augmin's partner proteins in living spermatocytes. This information in combination with studies of their dynamics will be crucial to fully dissect the molecular mechanism whereby Polo regulates Augmin's behaviour in male meiosis.

## 4. Concluding remarks

Bipolar spindle formation occurs through complementary centrosomal and centrosome-independent mechanisms. Here, we have investigated acentrosomal spindle formation in the specialized meiotic cells of the male germline. Our *Drosophila* spermatocyte studies reveal that despite a conservation of purpose and molecular components, fundamental aspects of spindle assembly vary between mitosis and male meiosis. Indeed, we find a common requirement for γ-tubulin and its Augmin targeting complex in acentrosomal MT nucleation and spindle formation, but note striking differences in the latter complex's distribution. This reflects a unique inhibitory mechanism that acts through Polo kinase. While the governing molecular events remain to be fully elucidated, by limiting Augmin and by extension γ-tubulin placement, this Polo-mediated pathway ultimately defines meiotic spindle density and architecture. Defining the mechanisms that underpin these differences and the advantages that they confer to meiotic spermatocytes will be an exciting future challenge.

## 5. Material and methods

### 5.1. Flies and husbandry

The following mutant and transgene expressing flies were used: *γ-tubulin23C^pi^* [31], *wacΔ 12* with the deficiency Df(3L)BSC125 [14], *β-tubulin56D::EGFP* [34], *EB1::EGFP* [49] and *polo^1^* with the deficiency Df(3L)RdgC-co2 [46]. Aurora B::mCherry expressing flies were generated by sequentially amplifying and ligating the Aurora B genomic region containing 1.5 kb of upstream regulatory sequence, including the promoter, the coding region and 0.5 kb of downstream sequence. A contig was assembled by standard restriction and ligation techniques and was sequenced prior to generating transgenic flies. Aurora B::mCherry behaves in a manner indistinguishable from the endogenous Aurora B protein as confirmed by localization studies. All flies were reared at 25°C according to standard methods.

### 5.2. Live cell imaging and microtubule repolymerization

Spermatocyte primary cultures were prepared by dissecting the testes from adult males in PBS. Following removal of unwanted tissues, the testes were transferred to an imaging chamber filled with Voltalef 10s oil as in Inoue *et al.* [34], but with the modification that the coverslips were now coated with the cell flattening agent concanavalin A (0.1 mg ml^−1^). Testes were ruptured under oil with individual cells assuming different extents of adherence. Flattened cells with co-planar structures were preferentially selected for imaging. Data were acquired on a Zeiss Axiovert 200 microscope equipped with a Perkin-Elmer RSIII spinning disk confocal head running Volocity software and using 63× (N.A. 1.4) or 100× (N.A. 1.4) lenses and a 2 × 2 bin. EGFP and mCherry were excited with 488 and 568 nm laser lines, respectively. At each 3–60 s time point, 3–7 z-sections at 0.5–1 µm increments were collected. For regrowth experiments, isolated testes were incubated for 20 min in PBS containing 1 μM colcemid in DMSO. Testes were similarly incubated in PBS supplemented with 1 μM BI 2536 (Axon Ligands) dissolved in DMSO to challenge Polo function. Drug-treated or DMSO solvent control exposed testes were transferred to oil prior to being ruptured and imaged. MT regrowth was initiated using the full intensity of a 50 W Hg lamp transmitted through a DAPI filter cube. To ensure colcemid inactivation, irradiation was performed for 4 s over multiple z-planes. Longer irradiation times did not increase MT regrowth efficiency. All acquisitions were performed at 25°C with a Zeiss TempControl 37-2 stage heater.

### 5.3. Quantification of live cell data

All quantifications were performed using Image J with the resultant data exported to Microsoft Excel for analysis or plot generation. To determine half-spindle elongation rates, datasets were imported and viewed as maximum intensity projections. Image series were aligned using the stackreg plugin with a rigid body transformation. Following calibration, object displacements were manually measured, plotted and velocity determined using a linear regression. To quantify fluorescence density, data series were imported into Image J and viewed as maximum intensity projections. Objects of interest were aligned as earlier. The mean fluorescence intensity for centrosomes and nuclei at each time point was measured with 5 and 10 μm diameter regions of interest, respectively. The values were multiplied by the area to give the signal's integrated fluorescence density. To correct for photobleaching, the background fluorescence was determined by applying the same method to MT-free regions of the cell. The signal-to-background ratio for each corrected object per time point was then calculated for 10 nuclei and 12 centrosomes per genotype. Standard error determination for fluorescence revealed that the wild-type consistently had the highest variation with an average of approximately 12% in the nuclei and approximately 3% at the centrosomes for each time point. The other genotypes varied on average 1–2% per time point irrespective of the area being analysed. These differences do not affect the nucleation trends. Thus, the plots reveal the average fluorescence ratios over time.

### 5.4. Immunofluorescence

Testes were dissected and incubated in PBS supplemented with the appropriate agent as above. Samples were fixed with −20°C methanol [34]. DMel-2 cells were maintained in Express Five serum-free media (Invitrogen) supplemented with l-glutamine, penicillin and streptomycin. Cells were plated on coverslips coated with 0.5 mg ml^−1^ concanavalin A and treated with 0.1% DMSO or 1 μM BI 2536 for 20 min in PBS prior to fixing with −20°C methanol. The following antibodies and concentrations were used: anti-CID at 1 : 100, anti-Dgt5 at 1 : 200 [11], anti-Dgt6 at 1 : 200 [21], anti-α-tubulin (DM1A at 1 : 100 and YL12 at 1 : 50) and anti-γ-tubulin (GTU-88) at 1 : 50. Images were acquired at 0.5 μm steps on a Zeiss 510 Meta confocal system using a 100× (N.A. 1.4) lens. Figures were processed and assembled in Adobe Photoshop.

## Supplementary Material

Electronic Supplementary Materials figures Legends

## Supplementary Material

Figure S1

## Supplementary Material

Figure S2

## Supplementary Material

Figure S3

## Supplementary Material

Figure S4
