# Supplementary material for: Differing requirements for Augmin in male meiotic and mitotic spindle formation in Drosophila
Source: Open Biol. 2014 May 14;4(5):140047. doi: 10.1098/rsob.140047 (PMC4042853; doi:10.1098/rsob.140047)
Supplement: Electronic Supplementary Materials figures Legends [file rsob140047supp1.docx]

**Electronic Supplementary Materials**

**figure S1. Kinetochore-derived MT structures share polarity with control spindles.** Images from time-lapse sequences of spermatocytes co-expressing EB1::EGFP to label growing MT plus ends and Aurora B::mCherry to identify kinetochore position. a) In non-treated control cells, EB1 comets are most concentrated at the centrosomes (*) consistent with extensive MT nucleation and growth at this site. EB1 foci are also found at; the persisting nuclear membranes (arrows), throughout the spindle including in the vicinity of the kinetochores (arrowheads) as well as at varying cytoplasmic positions reflecting the large aster size. Comets within the spindle are consistently oriented towards the equator, revealing the polarised nature of this structure. b) Images from a MT regrowth experiment. Following the UV pulse and MT regrowth, EB1 appears as a series of clustered punctae adjacent to the kinetochores. Arrowheads follow a single EB1 comet as it extends away from the kinetochore during MT elongation (102-126). A second comet (arrows) can be seen to interact with the kinetochore, corresponding with the chromosome’s displacement (195-282). Comets were always found within an arc of ~180° facing the chromosome during half-spindle formation. During later stages they sometimes radiated in several directions similar to centrosome-derived asters. Images are z-projections. Time in Sec relative to the UV pulse. Bar in low magnification and zoomed panels are 10 µm and 2 µm, respectively.

**figure S2. Augmin associates with kinetochores prior to spindle assembly.** An early prometaphase cell fixed and stained to reveal the distribution of centromeres (CID), microtubules and Augmin. Augmin decorates the kinetochores as shown by its close proximity to the centromeres. Although a single MT (arrowhead) can be seen to extend towards the cluster of centromeres, it does not contact any of them. Images are z-projections. Bar is 10 μm in low magnification and 2 μm in zoomed panels, respectively.

**figure S3. γ-tubulin and Augmin independently accumulate at the centrosomes.** a) γ-tubulin distribution in a *wac* mutant. The loss of Augmin activity does not preclude γ-tubulin’s decoration of the centrosomes. Note the normal appearance of the asters and spindle. b) Augmin distribution in a *γ-tubulin23C* mutant. Down-regulation of γ-tubulin does not prevent Augmin from being recruited to the centrosomes. However, the signal is less robust indicating a diminution of centrosome size in this mutant. Bars are 10 μm.

**figure S4. *γ-tubulin23C* mutants form unstable spindles.** a) Selected frames showing spindle morphogenesis in a *γ-tubulin23C* mutant expressing β-tubulin56D::EGFP to label MTs. Prominent asters emanate from the centrosomes (arrowheads) and fill the cytoplasm. Centrosomal MTs invade the nucleus during spindle formation (240) as occurs in the wild-type. As the nascent spindle becomes more dense and defined, the centrosomes move inwards and towards one another (240-1620). Time is in Sec relative to the onset of spindle formation. b) A *γ-tubulin23C* mutant cell fixed and stained to show CID to demarcate kinetochore position, microtubules and DNA. Large numbers of MTs surround the chromosomes between the two semi-collapsed centrosomes (arrowheads). Zoomed panel reveals that robust k-fibres are not conspicuous and kinetochores commonly contact the lateral face of proximal MTs. All images are z-projections. Bars are 10 μm except in zoomed panel where it its 2 μm.
