# Supplementary figures and images for: Differing requirements for Augmin in male meiotic and mitotic spindle formation in Drosophila
Source: Open Biol. 2014 May 14;4(5):140047. doi: 10.1098/rsob.140047 (PMC4042853; doi:10.1098/rsob.140047)

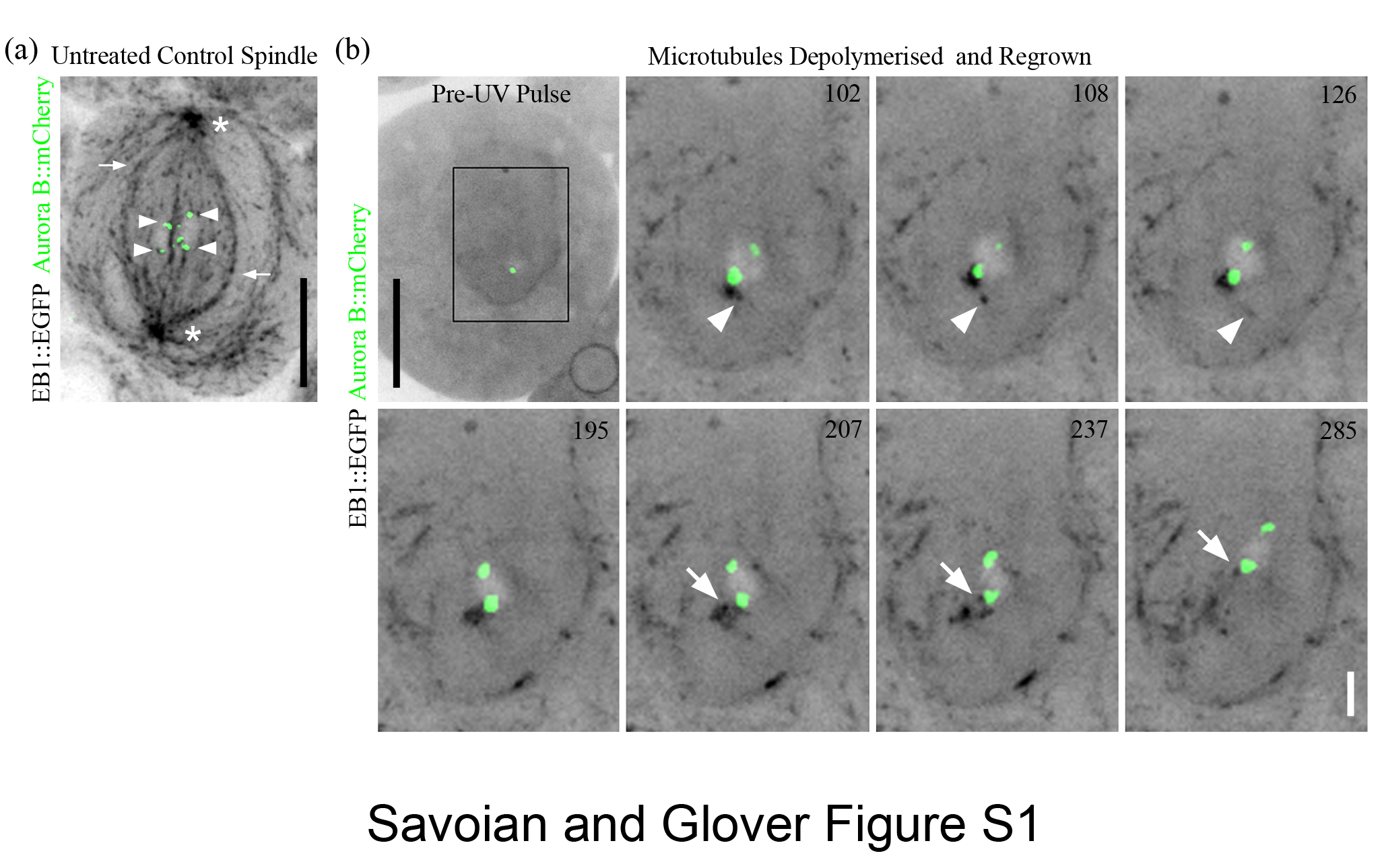

Supplement: Figure S1 [file rsob140047supp2.tif]

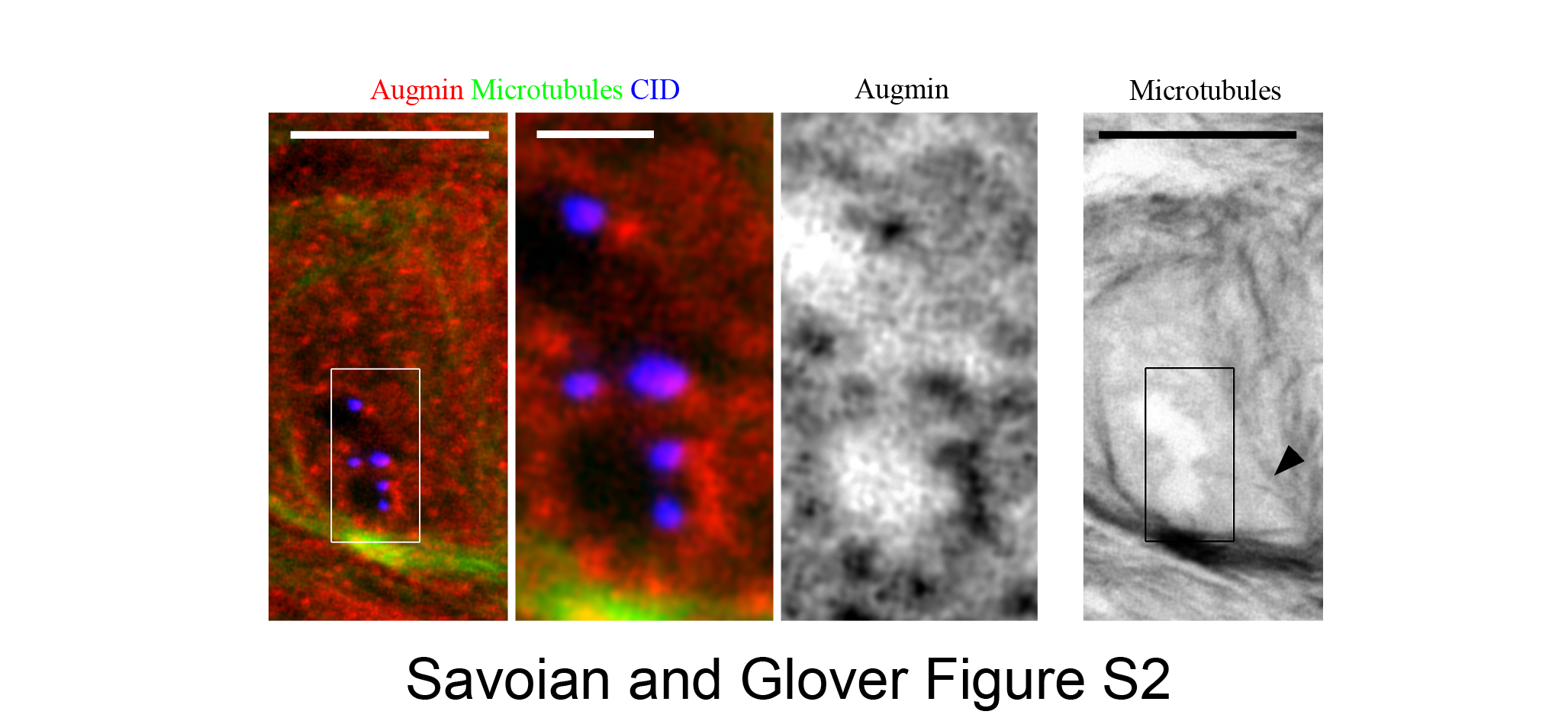

Supplement: Figure S2 [file rsob140047supp3.tif]

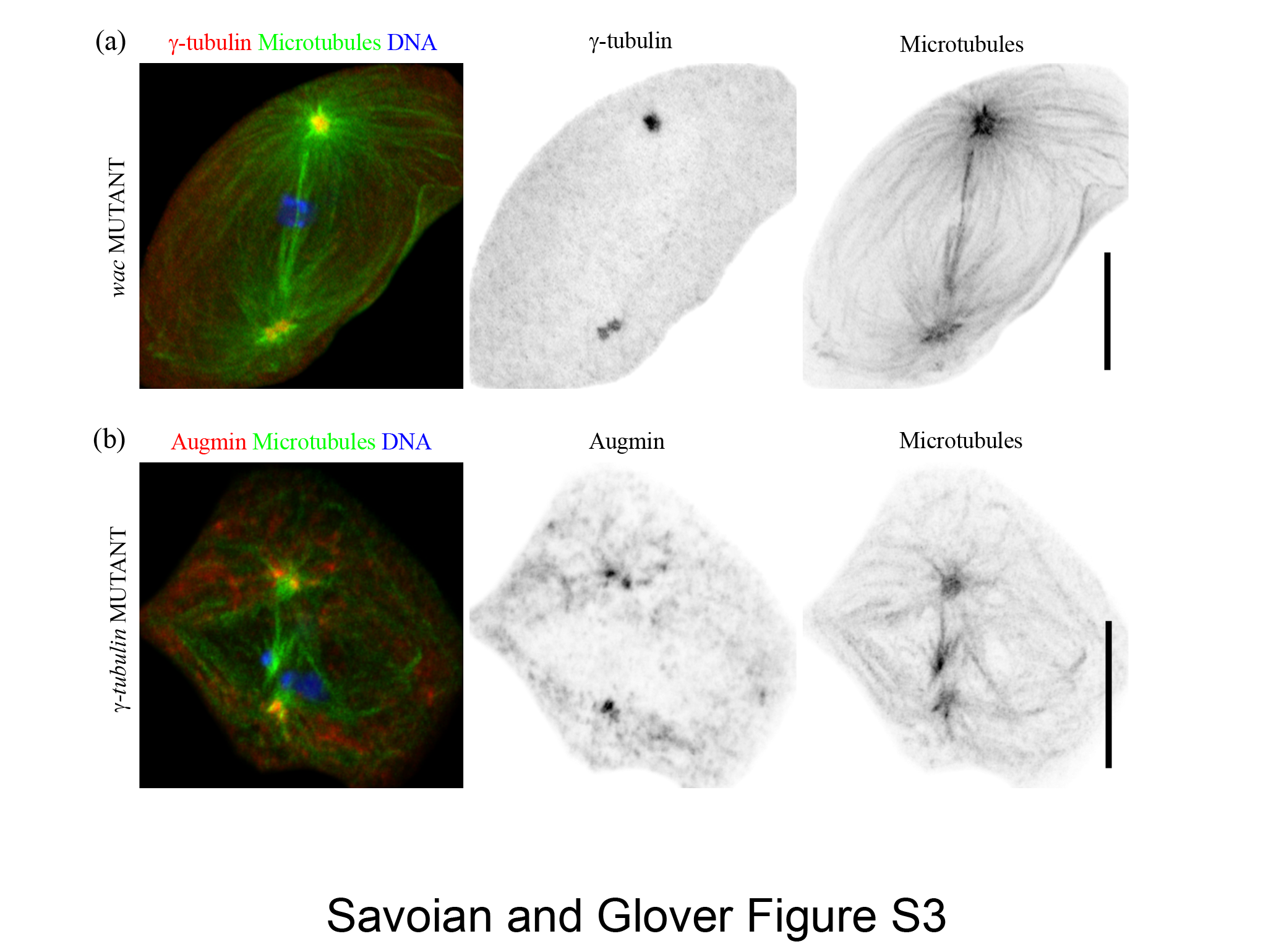

Supplement: Figure S3 [file rsob140047supp4.tif]

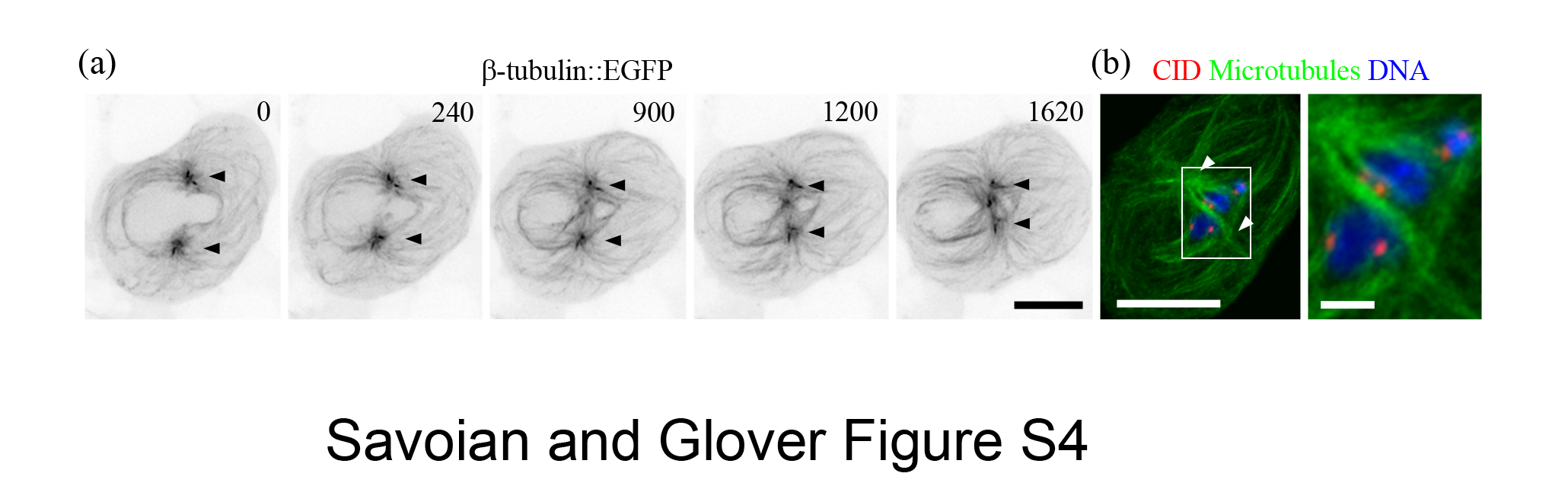

Supplement: Figure S4 [file rsob140047supp5.tif]
